# Distinct immunity dynamics of natural killer cells in mild and moderate COVID-19 cases during the Omicron variant phase

**DOI:** 10.3389/fimmu.2025.1594296

**Published:** 2025-05-12

**Authors:** Yukari Nishikawa, Kosuke Yamaguchi, Ma’arif Athok Shofiudin, Momone Mimura, Miyako Takata, Shu Mihara, Takeru Kawakami, Ayumu Doi, Risa Matsuda, Hiroyuki Kato, Ryo Okamoto, Kengo Mukuda, Naoki Kinoshita, Kensaku Okada, Tsuyoshi Kitaura, Masaki Nakamoto, Hisashi Noma, Yusuke Endo, Akira Yamasaki, Hiroki Chikumi

**Affiliations:** ^1^ Division of Infectious Diseases, School of Medicine, Faculty of Medicine, Tottori University, Yonago, Japan; ^2^ Department of Multidisciplinary Internal Medicine, Division of Respiratory Medicine and Rheumatology, Faculty of Medicine, Tottori University, Yonago, Japan; ^3^ Department of Pathobiological Science and Technology, Faculty of Medicine, Tottori University, Yonago, Japan; ^4^ Department of Interdisciplinary Statistical Mathematics, The Institute of Statistical Mathematics, Tachikawa, Japan; ^5^ Organisation for Research Institute and Promotion, Tottori University, Yonago, Japan

**Keywords:** COVID-19, Omicron variant, natural killer cells, cytokines, disease severity, ULBP2, NKG2D, TIGIT

## Abstract

**Background:**

The SARS-CoV-2 Omicron variant is associated with milder COVID-19 symptoms than previous strains. This study analyzed alterations in natural killer (NK) cell-associated immunity dynamics in mild and moderate COVID-19 cases during the Omicron phase of the COVID-19 pandemic.

**Methods:**

We conducted a retrospective observational cohort study of patients aged ≥16 with confirmed SARS-CoV-2 infection who were hospitalized at Tottori University Hospital between January 2022 and May 2022. A total of 27 patients were included in the analysis. Of these, 11 and 16 were diagnosed with mild and moderate COVID-19, respectively, based on the Japanese COVID-19 clinical practice guideline. Peripheral blood NK cell subsets and surface markers, including the activating receptor NKG2D and the inhibitory receptor TIGIT, as well as serum levels of 24 immunoregulatory markers, such as cytokines and cytotoxic mediators, were measured at admission and recovery. In addition, to explore immune patterns associated with disease severity, differences in 24 serum markers and soluble UL16-binding protein 2 (sULBP2) at the clinically most symptomatic time point during hospitalization were visualized using a volcano plot and analyzed with Spearman’s rank correlation analysis and principal component analysis (PCA).

**Results:**

Patients with mild COVID-19 exhibited expanded subsets of unconventional CD56^dim^CD16^-^ NK cells with elevated NKG2D expression and lower levels of cytotoxic mediators (granzyme A, granzyme B, and granulysin). In contrast, patients with moderate disease exhibited NK cell exhaustion, characterized by upregulation of TIGIT, along with increased levels of NK cell-associated cytokines and cytotoxic mediators. The volcano plot identified that the patients with moderate COVID-19 exhibited significantly elevated IL-6 and sULBP2 levels. Spearman’s rank correlation analysis revealed that IL-6, IFN-γ, soluble Fas, and CXCL8 were correlated with increased sULBP2. The PCA identified distinct clusters based on disease severity.

**Conclusions:**

The results of study highlight the differences in NK cell-associated immune alterations between mild and moderate COVID-19 cases. Elevated IL-6 and sULBP2 levels, along with their correlations with inflammatory mediators, reflects differences in immune response based on disease severity. These findings provide insight into the immune response to infection caused by the Omicron variant of SARS-CoV-2 and improve our understanding of its immunological features.

## 1 Introduction

COVID-19, an acute respiratory infection caused by SARS-CoV-2, spread rapidly worldwide from late 2019 to 2023, leading to a global pandemic and posing severe public health challenges in numerous countries (1, 2). The clinical manifestations of COVID-19 typically include fever, fatigue, non-productive cough, headache, diarrhea, sore throat, and olfactory and gustatory disturbances. However, in some patients, the disease progresses to severe forms, developing pneumonia and respiratory failure. In critical cases, COVID-19 may evolve into acute respiratory distress syndrome, potentially resulting in fatal outcomes (3). The SARS-CoV-2 Omicron variant (B.1.1.529), which emerged in December 2021, exhibited higher transmissibility and a shorter incubation period than the previously detected strains. Moreover, it is associated with milder cases of COVID-19 than the delta variant, which was the predominant strain prior to emergence of the Omicron variant (4). However, the host immune response factors contributing to these changes in disease severity during the Omicron-dominant phase remain unclear.

Natural killer (NK) cells, a crucial component of the innate immune system, play a pivotal role in defending against viral infections. Prior to the emergence of the Omicron variant, studies indicated that patients with COVID-19 exhibited decreased NK cell count from the early stages of the disease, which correlated with disease severity, with recovery observed as patients convalesced (5). Furthermore, NK cell activation during the acute phase of SARS-CoV-2 infection has been reported to be accompanied by the upregulation of inhibitory checkpoint receptors, initiating a regulatory program (6). However, data on functional changes and alterations in NK cell surface receptors throughout the clinical course of mild-to-moderate COVID-19, which became predominant during the Omicron wave, remain scarce. Moreover, the mechanisms by which viral infections modulate these receptors are not well understood. NK cell activity is finely tuned by the balance between activating receptors, such as DNAM-1 and NKG2D, and inhibitory receptors, such as LAG-3, TIGIT, and TIM-3, which are expressed on NK cell surfaces. Understanding the intricate interplay between viral infections and these receptors is crucial for elucidating the complex dynamics of NK cell responses to COVID-19.

UL-16 binding protein 2 (ULBP2), a ligand for the activating receptor NKG2D on NK cells, is induced on the surfaces of host cells during viral infection, leading to NK cell activation (7). Previous studies have shown that ULBP2 undergoes shedding from the host cell surface and is released into the serum as soluble ULBP2 (sULBP2). Furthermore, *in vitro* studies have demonstrated a correlation between ULBP2 expression on cultured cell surfaces and sULBP2 concentrations in cell supernatants (8). Based on these findings, we consider that measuring serum sULBP2 levels could serve as an indirect indicator of ULBP2 expression on cell surfaces, potentially aiding in the assessment of the impact of viral infection on NK cells.

In this study, we analyzed alterations in NK cell subsets within peripheral blood mononuclear cells (PBMCs), along with the surface markers NKG2D and TIGIT, in patients with mild-to-moderate COVID-19 during the Omicron phase of the pandemic, and examined their associations with disease severity. Furthermore, we investigated temporal fluctuations in 24 markers, including cytokines, cytotoxic mediators, and soluble markers, associated with NK cell, CD8^+^ T cell, and B cell functions and analyzed their correlations with sULBP2 levels. Through this approach, we sought to gain insight into the mechanisms by which viral infection modulates NK cell functionality.

## 2 Materials and methods

### 2.1 Ethical considerations

This retrospective observational cohort study was conducted at Tottori University Hospital in accordance with the ethical principles outlined in the Declaration of Helsinki. The research protocol was approved by the Institutional Review Board (IRB) of Tottori University Faculty of Medicine (approval number: 21A168). Written informed consent was obtained from patients at the time of hospital admission if additional blood sampling was required. For cases where additional blood sampling was not required, consent was obtained using an opt-out method, as approved by the IRB and in accordance with the Ethical Guidelines for Medical and Biological Research Involving Human Subjects (9). The study details, including its purpose, procedures, and the right to decline participation, were publicly accessible on hospital noticeboards. Patients or their representatives had the option to decline the use of their existing biological specimens and clinical information in this study by contacting the research team directly.

### 2.2 Patient selection

Patients aged 16 years or older who were hospitalized for COVID-19 between January 2022 and May 2022 were enrolled in this study. COVID-19 diagnosis was confirmed by a positive PCR assay using nasal swabs or saliva specimens. Genomic surveys conducted by the regional government during the study period indicated that the Omicron BA.1 and BA.2 variants were predominance (10, 11). The severity of COVID-19 was classified as mild or moderate based on the Japanese clinical practice guideline (12). Severe cases were excluded from this study. Details of the patient selection process are provided in the **Supplementary Methods** and **Supplementary Figure 1**. Data on the patient demographics, comorbidities, COVID-19 vaccination status, treatment details, and laboratory findings were obtained from their medical records. Healthy controls were not included, as the study aimed to compare immune responses between mild and moderate COVID-19 cases during the Omicron-dominant phase. To compensate for the lack of healthy controls, longitudinal analyses between admission and recovery were used to assess immune changes over time.

### 2.3 Blood sample collection and processing

Blood samples for the isolation of PBMCs and serum separation were collected from the patients at two primary time points (admission and recovery), with an additional time point analyzed in selected cases who experienced clinical worsening during hospitalization. For isolation of PBMCs, blood was collected in EDTA tubes, stored at 4°C, and processed within 24 hours. The PBMCs were isolated using Ficoll-Paque density gradient media (Merck KGaA) and SepMate™ PBMC Isolation Tubes (STEMCELL Technologies). The isolated PBMCs were then cryopreserved in serum-free media (Bambanker™, GC Lymphotec Inc.) at −80°C. For serum separation, blood was collected in serum separation tubes, allowed to clot at room temperature for 30 minutes, and centrifuged at 1,800 × g for 10 minutes. The obtained serum was kept at 4°C and stored at −80°C within 24 hours. All subsequent experiments involving PBMCs and serum were performed using standardized protocols to ensure consistency across groups.

### 2.4 Phenotype characterization

Thawed PBMCs were washed and resuspended in phosphate-buffered saline (PBS) (−) (164-23551, Fujifilm Wako Chemicals) containing 0.5% bovine serum albumin (BSA) and 2 mM EDTA. Fc receptor blocking was performed by incubating cells with Human TruStain FcX™ (Fc receptor blocking solution, 422302, BioLegend) for 10 minutes at 4°C. Cells were then stained with a panel of fluorochrome-conjugated antibodies, including: PerCP/Cyanine 5.5-conjugated anti-CD3 antibody (300327, BioLegend), PE/Dazzle-conjugated anti-CD8α antibody (300929, BioLegend), PE/Cyanine 7-conjugated anti- CD56 antibody (362510, BioLegend), Brilliant Violet 510-conjugated anti-CD16 antibody (302047, BioLegend), Alexa Fluor 488-conjugated anti-CD27 antibody (393203, BioLegend), Brilliant Violet 421-conjugated anti-TIGIT antibody (372709, BioLegend), and APC-conjugated anti-NKG2D antibody (320807, BioLegend). Live/dead cell discrimination was performed using Fixable Viability Dye eFluor™ 780 (65-0865-14, eBiosciences). After staining, cells were washed twice, filtered through a 40 μm nylon mesh, and acquired using a Cytoflex flow cytometer (Beckman Coulter). Data analysis was performed with FlowJo™ version 10 (Becton Dickinson). The gating strategies are shown in **Supplementary Figures 2** and **3A**.

### 2.5 Measurement of serum concentrations of cytokines, cytotoxic mediators, and soluble markers

Serum concentrations of cytokines, cytotoxic mediators, and soluble markers were measured using LEGENDplex™ multi-analyte flow assay kits, including the human CD8/NK Panel (741065, BioLegend), COVID-19 Cytokine Storm Panel (741091, BioLegend), and Human B cell Activator Panel (740535, BioLegend). These panels were used to quantify IL-2, IL-4, IL-6, IL-10, IL-17A, TNF-α, IFN-γ, soluble Fas (sFas), soluble FasL (sFasL), granzyme A, granzyme B, perforin, granulysin, IFN-α2, CXCL8 (IL-8), CCL2 (MCP-1), G-CSF, IL-7, IL-1RA, CXCL10 (IP-10), MIP-1α (CCL3), APRIL, BAFF, and soluble CD40L (sCD40L). Serum sULBP2 concentrations were measured using the Human ULBP2 ELISA Kit (ab288172, Abcam). All measurements were performed strictly according to the manufacturer’s instructions provided with the experimental kit.

### 2.6 Statistical analysis

Patient characteristics were compared using the Mann–Whitney U test (age) and Fisher’s exact test (sex, vaccination status, comorbidities, and COVID-19 treatment). Within-group comparisons between admission and recovery were performed using the Wilcoxon signed-rank test, while between-group comparisons were assessed using the Mann–Whitney U test. Analyses were performed using available data for each outcome, excluding patients with missing values. P < 0.05 was considered as statistically significant. To visualize differences in serum marker levels between mild and moderate cases at the clinically most symptomatic time point during hospitalization, a volcano plot was generated. Fold changes were calculated as the ratio of the mean values (moderate group/mild group), and statistical significance was assessed using the Mann–Whitney U test. False discovery rate (FDR) correction was applied using the two-stage linear step-up procedure of Benjamini, Krieger, and Yekutieli (13), with an adjusted significance threshold of q < 0.05. The pairwise correlation coefficients among all cytokines and cytotoxic mediators were calculated using Spearman’s rank correlation, and the resulting correlation matrix was used to identify significant associations between immune markers. Simple linear regression analysis was performed to assess the relationships between key cytokines and immune parameters. Principal component analysis (PCA) was performed to explore the relationships among the 24 serum markers plus sULBP2 (25 parameters in total) and identify key patterns of variance in the dataset. Serum marker values were log-transformed and standardized (mean = 0, standard deviation = 1) before PCA. The number of principal components (PCs) to retain was determined using parallel analysis, and variable loadings were examined to interpret the biological significance of each PC. A color-mapped PCA score plot was generated to visualize the distribution of patients based on their PCA scores. All analyses were performed using GraphPad Prism (version 10.0.2).

## 3 Results

### 3.1 Patient demographics and clinical characteristics

A total of 27 patients diagnosed with COVID-19 were included in this study. Patient characteristics are summarized in **Table 1**. Of the 27 patients, 11 were in the mild group and 16 were in the moderate group. Blood cell analysis was conducted for 26 cases due to technical issues with one moderate case, while serum analysis was performed for all 27 cases. The mean age of the patients differed significantly between the two groups: the mean age of the patients in the mild group was 34 years (IQR: 28.5–66.5), whereas that of patients in the moderate group was 74 years (IQR: 56.5–79.0). Sex distribution and vaccination rates were comparable between the groups. Sixteen comorbidities were examined in this study (**Table 1**), and no significant differences were observed in any of the underlying conditions between the two groups.

**Table 1:** Patient characteristics.

Clinical Characteristics	Mild group	Moderate group	p value
Cases	11	16	
Mean of age (IQR)	34 (28.5-66.5)	75 (58.3-79.5)	< 0.001
Male (%)	4 (36.4%)	10 (62.5%)	0.252
Vaccinated (%)	9 (81.8%)	9 (56.3%)	0.231
Comorbidities			
COPD	0	1 (6.3%)	> 0.999
DM	1 (9.1%)	5 (31.3%)	0.350
HL	2 (18.2%)	5 (31.3%)	0.662
HT	3 (27.3%)	5 (31.3%)	> 0.999
CKD	0	6 (37.5%)	0.054
Malignancy	4 (36.4%)	3 (18.8%)	0.391
Obesity (BMI > 30 kg/m^2^)	0	6 (37.5%)	0.054
Smoking experience	0	3 (18.8%)	0.248
Immunosuppressant	2 (18.2%)	6 (37.5%)	0.405
Cerebrovascular disease	1 (9.1%)	1 (6.3%)	> 0.999
Heart failure	0	2 (12.5%)	0.499
Interstitial pneumonia	0	1 (6.3%)	> 0.999
Bronchiectasis	1 (9.1%)	0	0.407
Tuberculosis	0	1 (6.3%)	> 0.999
Alcoholic hepatitis	1 (9.1%)	1 (6.3%)	> 0.999
Mood disorders	0	1 (6.3%)	> 0.999
Treatment for COVID-19			
Dexamethasone	0	13 (81.3%)	< 0.001
Remdesivir	0	12 (75.0%)	< 0.001
Baricitinib	0	1 (6.3%)	> 0.999
Sotrovimab	4 (36.4%)	1 (6.3%)	0.125
Molnupiravir	1 (9.1%)	3 (18.8%)	0.624

Patient characteristics were compared using the Mann–Whitney U test for age and Fisher’s exact test for sex, vaccination status, comorbidities, and COVID-19 treatment. COPD, chronic obstructive pulmonary diseases; DM, diabetes mellites; HL, hyperlipidemia; HT, hypertension; CKD, chronic kidney disease.

Treatment for COVID-19 with dexamethasone and remdesivir was significantly more frequent in the moderate group than in the mild group. All mild cases recovered without exacerbation, and admission was therefore considered the clinically most symptomatic time point during hospitalization. Among the 16 moderate cases, 14 also showed no clinical worsening after admission, and admission was likewise considered the clinically most symptomatic time point. For the remaining two moderate cases, this timepoint was defined as the time of clinical exacerbation after admission. The number of days since symptom onset at each timepoint is summarized in **Table 2**.

**Table 2:** Days since symptom onset (DSSO) at each blood sampling timepoint.

Severity	Timepoint	DSSO (median, IQR)
Mild	Admission	2 (1–3)
	Recovery	9 (8–11)
Moderate	Admission	5 (2–7)
	Clinically most symptomatic time point during hospitalization	5 (3–7)
	Recovery	13 (11–17)

“Clinically most symptomatic time point during hospitalization” refers to the timepoint at which the patient’s symptoms were most pronounced during the hospital stay. For all 11 mild cases and 14 of the 16 moderate cases, admission was considered the clinically most symptomatic time point. For the remaining two moderate cases, it was defined as the time of clinical exacerbation after admission. Accordingly, the values shown for this timepoint in the moderate group include both admission and post-admission sampling, depending on the individual case.

### 3.2 Differences in laboratory findings between the mild and moderate COVID-19 cases at admission

**Figure 1:**
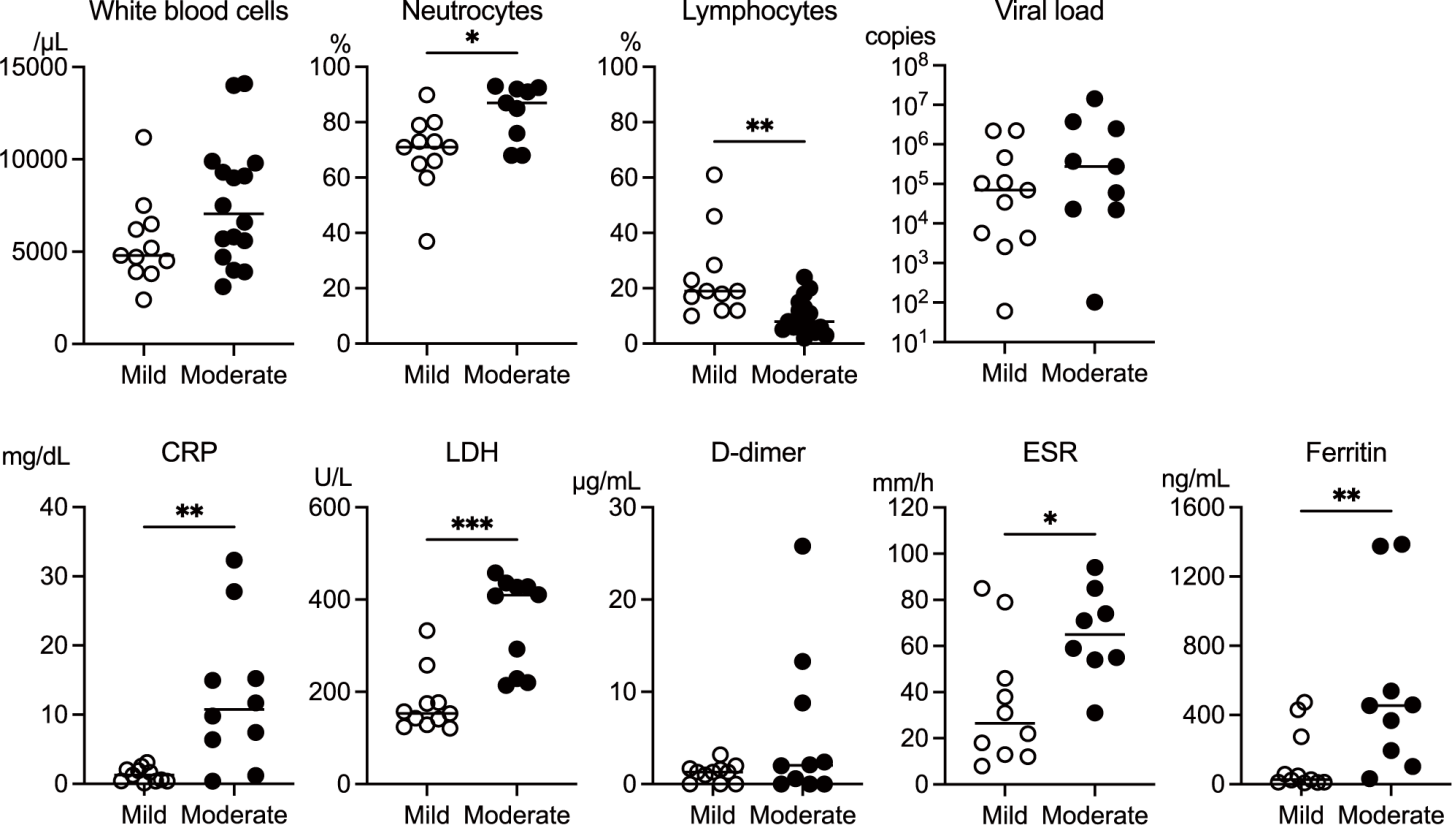
Comparison of laboratory parameters measured at the time of admission between the mild and moderate COVID-19 groups. The data on white blood cell count, the ratio of neutrophils and lymphocytes, viral load, erythrocyte sedimentation rate (ESR), and C-reactive protein (CRP), lactate dehydrogenase (LDH), d-dimer, and ferritin levels were extracted from the medical records of the patients in the mild (n = 11) and moderate (n = 16) groups. Extraction of the data extracted from electronic medical records limited the sample size. The median values were compared using the Mann–Whitney test. The white circles and black circles represent individual patient values for the mild group and moderate group, respectively. The horizontal bars indicate the median values. P values are indicated as follows: *p < 0.05, **p < 0.01, ***p < 0.001. Non-significant differences are not marked.

Laboratory findings at admission differed significantly between the mild and moderate groups (**Figure 1**). The test parameters were extracted from electronic medical records, and some values were missing depending on admission circumstances. The analysis revealed notable differences in leukocyte profiles and inflammatory markers between the two groups. The mild group had a significantly higher proportion of lymphocytes, whereas the moderate group exhibited significantly elevated neutrophil percentages. Inflammatory markers, including C-reactive protein (CRP), lactate dehydrogenase (LDH), erythrocyte sedimentation rate (ESR), and ferritin levels, were markedly elevated in the moderate group. Notably, viral load at admission did not differ significantly between the groups. These findings suggest that the patients with moderate COVID-19 exhibited a more pronounced inflammatory response, predominantly mediated by neutrophils, than those with mild COVID-19.

### 3.3 Temporal changes in NK cell subsets and the expression profiles of NKG2D and TIGIT in patients with mild and moderate COVID-19

**Figure 2:**
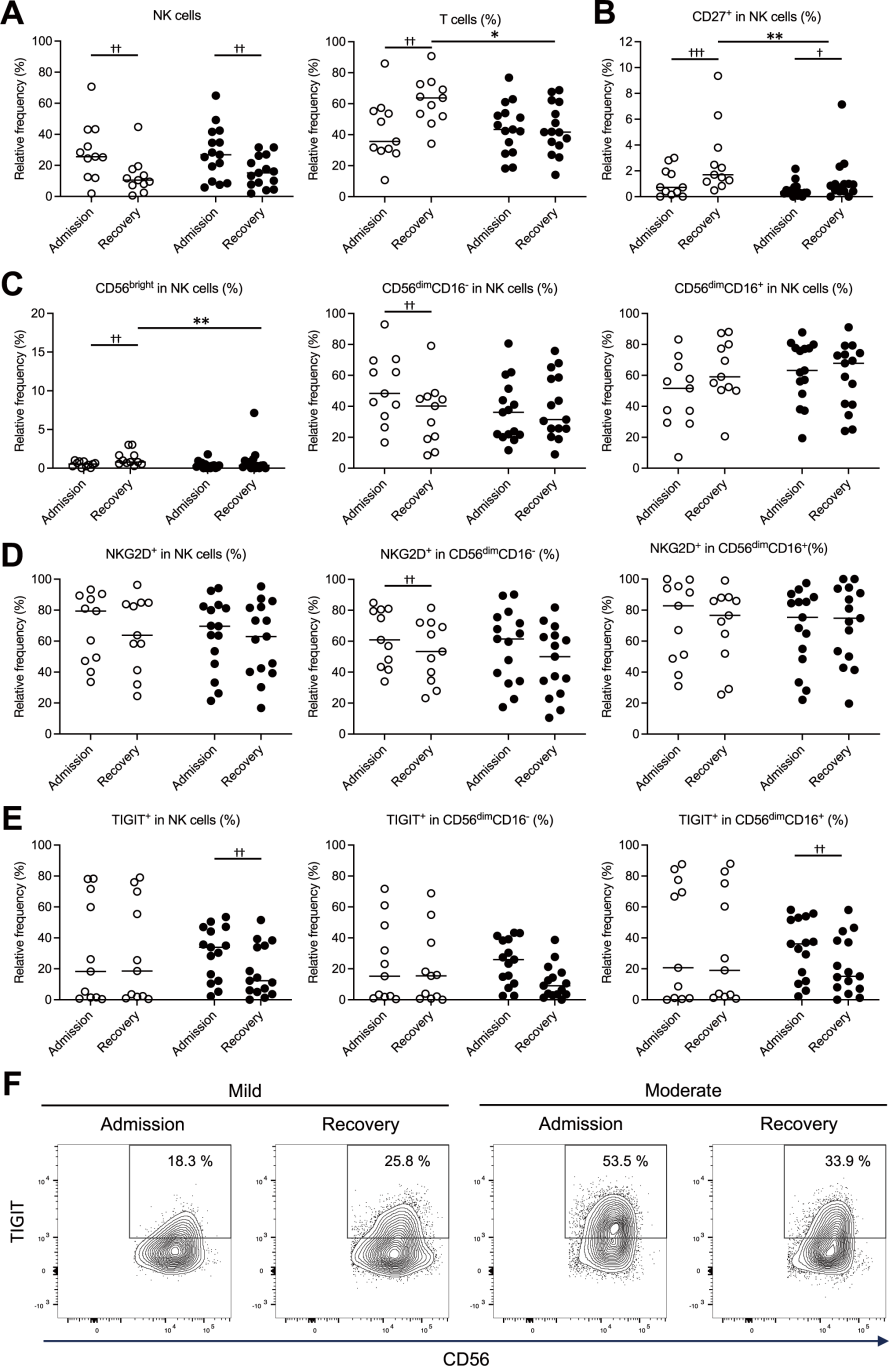
Temporal changes in NK cell subsets and expression profiles of NKG2D and TIGIT in the mild and moderate COVID-19 groups. NK cell profiles measured at the time of admission and during recovery were compared between the mild (n = 11) and moderate (n = 15) groups, highlighting differences associated with disease severity. **(A)** Proportions of NK cells (CD3^-^CD56^+^) and T cells (CD3^+^CD56^-^) within peripheral blood mononuclear cells (PBMCs). **(B)** Proportions of CD27^+^ cells in NK cells. **(C)** Proportions of NK cell subsets (CD56^bright^, CD56^dim^CD16^-^, CD56^dim^CD16^+^) in NK cells. **(D)** Proportions of all the NK cells and their subsets (CD56^dim^CD16^-^, CD56^dim^CD16^+^) expressing NKG2D on the cell surface. **(E)** Proportions of all the NK cells and their subsets (CD56^dim^CD16^-^, CD56^dim^CD16^+^) expressing TIGIT on the cell surface. **(F)** Representative counter plots corresponding to the data in **(E)**, illustrating the proportions of TIGIT-expressing cells among all the NK cells. In **(A–E)**, the white and black circles represent individual patient data for the mild group and the moderate group, respectively, and the horizontal bars indicate median values. The Wilcoxon signed-rank test was used for comparisons between the admission and recovery phases, and the Mann–Whitney U test for comparisons between disease severity groups. Significance levels were set as follows: †p < 0.05, ††p < 0.01, †††p < 0.001 for the Wilcoxon signed-rank test, *p < 0.05, **p < 0.01, for the Mann–Whitney U test. Non-significant differences are not marked.

We examined temporal changes in the proportions of NK cells, T cells, CD8^+^ T cells, and NK cell subsets (CD56^bright^ NK cells, CD56^dim^CD16^-^ NK cells, CD56^dim^CD16^+^ NK cells) from admission to recovery in patients with mild and moderate COVID-19. Additionally, we analyzed the proportions of cells expressing CD27, a differentiation marker of NK cells. At the time of admission, the patients in the mild group showed a significantly higher proportion of NK cells and a markedly decreased proportion of T cells in total PBMCs (**Figure 2A**). Among the expanded NK cells, the proportions of CD27^+^ NK cells and CD56^bright^ NK cells were significantly reduced (**Figure 2B**), while the proportion of CD56^dim^CD16^-^ NK cells was significantly increased (**Figure 2C**). The moderate group showed a similar trend in the proportions of total NK cells (**Figure 2A**) and CD27^+^ NK cells (**Figure 2B**). However, in the moderate group, the proportions of CD56^bright^ NK cells, CD56^dim^CD16^-^ NK cells, and CD56^dim^CD16^+^ NK cells within the NK cell population did not differ significantly between admission and recovery (**Figure 2C**). These findings suggest that although the patients with mild COVID-19 exhibited suppression of T cells and immature NK cell subsets (CD27^+^ NK cells and CD56^bright^ NK cells) at the time of admission, their overall NK cell proportions in these patients increased, primarily driven by a substantial rise in the proportions of CD56^dim^CD16^-^ NK cells.

The activation status of NK cells was assessed by analyzing the expression of NKG2D, an activating receptor, and TIGIT, an inhibitory receptor, on their surfaces. Patients in the mild group showed significantly higher NKG2D expression on CD56^dim^CD16^-^ NK cells at the time of admission (**Figure 2D**). In contrast, patients in the moderate groups displayed significantly elevated TIGIT expression in NK and CD56^dim^CD16^+^ NK cells upon admission (**Figures 2E, F**). These findings suggest that in mild COVID-19, increased expression of activating receptors on CD56^dim^CD16^-^ NK cells may contribute to NK cell activation, whereas in moderate disease, higher expression of inhibitory receptors on CD56^dim^CD16^+^ NK cells may indicate a shift toward NK cell inhibition.

### 3.4 Relationship between TIGIT and NKG2D expression in NK and CD8^+^ T cells in mild and moderate COVID-19

**Figure 3:**
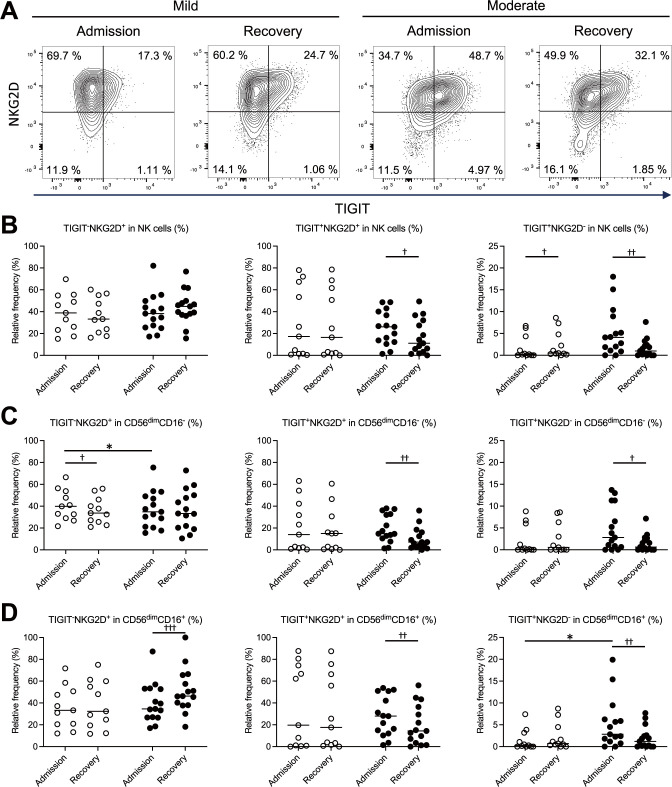
Relationship between TIGIT and NKG2D expression in NK cells in patients with mild and moderate COVID-19. Flow cytometry of PBMCs was performed to generate two-dimensional plots of TIGIT and NKG2D expression on NK cells (CD3^-^CD56^+^). The proportions of TIGIT^-^NKG2D^+^, TIGIT^+^NKG2D^+^, and TIGIT^+^NKG2D^-^ cells at the time of admission and during recovery were compared between patients in the mild (n = 11) and moderate (n = 15) groups, highlighting differences related to disease severity. **(A)** Representative contour plots of the total NK cells, with TIGIT on the x-axis and NKG2D on the y-axis. **(B–D)** Proportions of TIGIT^-^NKG2D^+^, TIGIT^+^NKG2D^+^, TIGIT^+^NKG2D^-^ cells among all the NK cells **(B)**, the CD56^dim^CD16^-^ NK cell subset **(C)**, and the CD56^dim^CD16^+^ NK cell subset **(D)**. In **(B–D)**, the white circles and black circles represent individual patient data for the mild group and moderate group, respectively, and the horizontal bars indicate median values. The Wilcoxon signed-rank test was used for comparisons between the admission and recovery phases, and the Mann–Whitney U test for comparisons between disease severity group. Significance levels were set as follows: †p < 0.05, ††p < 0.01, †††p < 0.001 for the Wilcoxon signed-rank test, *p < 0.05 for the Mann–Whitney U test. Non-significant differences are not marked.

The expression levels of TIGIT and NKG2D at the time of admission and during recovery were measured by flow cytometry, visualized using two-dimensional plots, and compared between the mild and moderate groups. In the three examined subsets—total NK cells (**Figures 3A, B**), CD56^dim^CD16^-^ NK cells (**Figure 3C**), and CD56^dim^CD16^+^ NK cells (**Figure 3D**) —the proportions of TIGIT^+^NKG2D^+^ and TIGIT^+^NKG2D^-^ cells were elevated in the moderate group at the time of admission. Conversely, in the mild group, the proportion of TIGIT^+^NKG2D^-^ cells among total NK cells was lower. Furthermore, a comparison between the mild and moderate groups at the time of admission revealed no significant differences in the proportions of TIGIT^+^NKG2D^+^ cells across the three subsets (**Figures 3B–D**). However, the proportion of TIGIT^+^NKG2D^-^ cells was significantly higher in CD56^dim^CD16^+^ NK cells in the moderate group (**Figure 3D**). These findings suggest that in mild COVID-19, NK cells may retain NKG2D expression while expressing TIGIT, enabling them to sustain activation despite progressing toward exhaustion. In contrast, in moderate COVID-19, TIGIT-expressing NK cells exhibited reduced NKG2D expression, potentially leading to increased exhaustion and decreased activation.

A similar pattern was observed in CD8^+^ T cells. The patients in the moderate groups showed a significantly higher proportion of TIGIT^+^NKG2D^-^ cells in at the time of admission than patients in the mild group (**Supplementary Figures S3A–C**). Additionally, an analysis of CD27 and TIGIT expression on CD8^+^ T cells revealed that the patients in the moderate groups exhibited a significantly elevated proportion of CD27^-^TIGIT^+^ cells on admission compared to both patients in the mild group and their own recovery phase (**Supplementary Figures S3D, E**). The increase in these cell populations may reflect changes in the activation status or differentiation stage of T cells.

### 3.5 Cytokine and cytotoxic mediator profiles of NK cell-mediated immunity in patients with mild and moderate COVID-19

**Figure 4:**
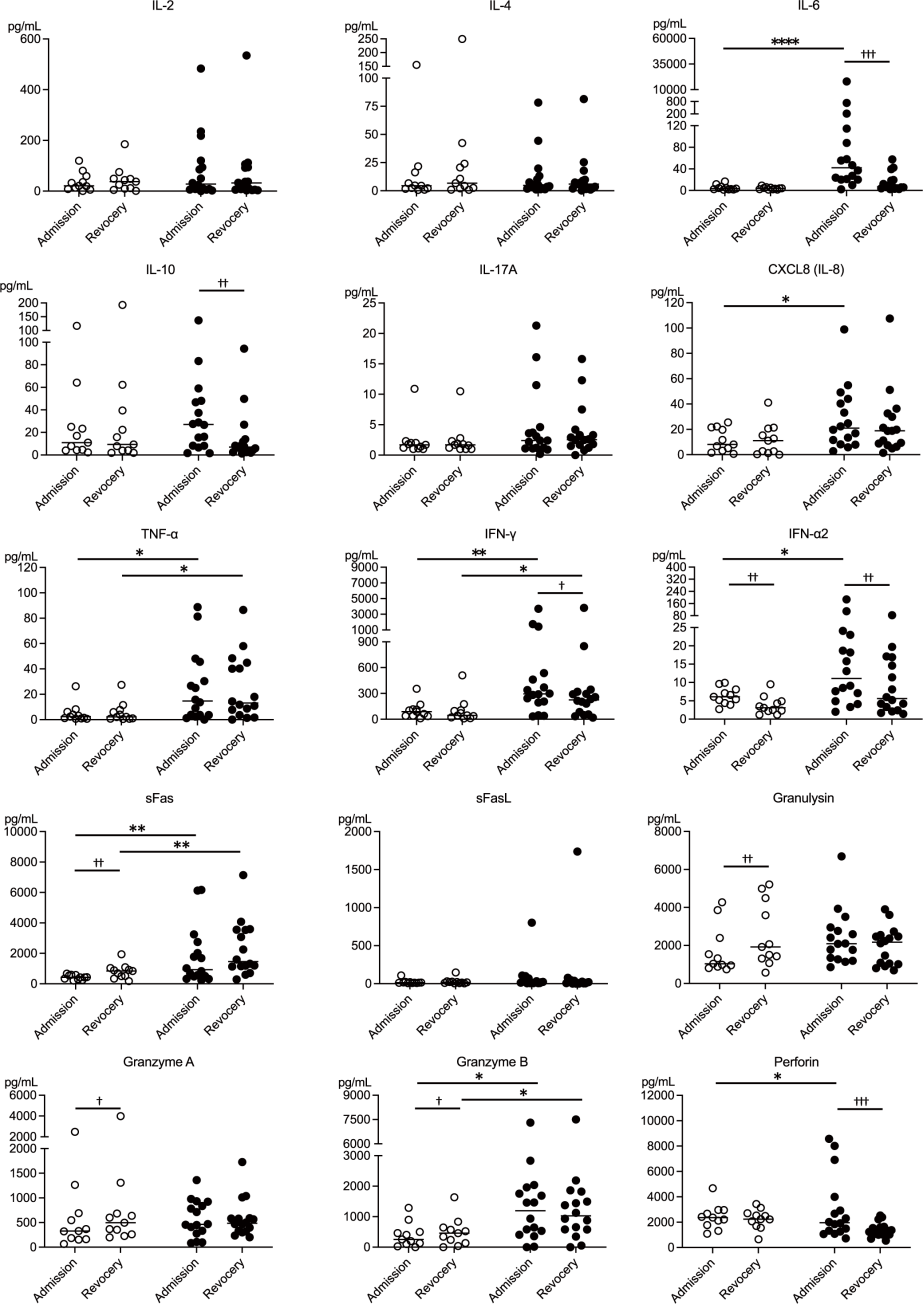
Cytokine and cytotoxic mediator profiles of NK cell-mediated immunity during mild and moderate COVID-19. Serum levels of 15 NK cell-related cytokines and cytotoxic mediators at the time of admission and during recovery were compared between the mild (n = 11) and moderate (n = 16) groups, highlighting differences related to disease severity. The white circles and black circles represent individual patient data for the mild group and moderate group, respectively, and the horizontal bars indicate median values. Wilcoxon signed-rank test was used for comparisons between the admission and recovery phases, and the Mann–Whitney U test for comparisons between disease severity groups. Significance levels were set as follows: †p < 0.05, ††p < 0.01, †††p < 0.001 for the Wilcoxon signed-rank test, *p < 0.05, **p < 0.01, ****p < 0.0001 for the Mann–Whitney U test. Non-significant differences are not marked.

In addition to flow cytometry analysis of PBMCs, serum levels of 24 markers, including cytokines, cytotoxic mediators, and soluble markers, were measured at admission and recovery. Data on 16 cytokines and cytotoxic mediators associated with NK cell function are presented in **Figure 4**. The moderate group exhibited significantly higher levels of inflammatory cytokines, including IL-6, CXCL8 (IL-8), TNF-α, IFN-γ, and IFN-α2, compared to the mild group. TNF-α and IFN-γ levels remained elevated in the moderate group during the recovery. Cytokine levels between admission and recovery phases showed no significant differences in inflammatory cytokines among mild cases. However, patients in the moderate group showed significantly higher IL-6, IFN-γ, and IFN-α2 levels on admission than during recovery. Additionally, patients in the moderate group showed significantly higher levels of the anti-inflammatory cytokine IL-10 on admission than during recovery. These findings suggest that, compared to the patients with mild COVID-19, the patients with moderate COVID-19 exhibited a significant increase in both inflammatory and anti-inflammatory cytokines, with elevated levels persisting into the recovery phase.

Patients in the mild group showed lower levels of sFas, which is associated with host cell apoptosis, at the time of admission than during the recovery phase. In contrast, patients in the moderate group exhibited higher sFas levels both on admission and during recovery than those in the mild group. These findings suggest that the susceptibility of host cells to apoptosis may have been suppressed in the patients with mild COVID-19 and enhanced in those with moderate COVID-19. Regarding cytotoxic mediators secreted by NK cells, the patients in the mild group exhibited decreased levels of granulysin, granzyme A, and granzyme B at the onset of the disease. Conversely, patients in the moderate group showed elevated perforin levels at disease onset. These results indicate that the release of cytotoxic mediators may have been suppressed in the patients with mild COVID-19 and enhanced in those with moderate COVID-19. The profiles of the remaining eight markers are shown in **Supplementary Figure S4**.

### 3.6 Elevated sULBP2 levels in moderate COVID-19 cases

**Figure 5:**
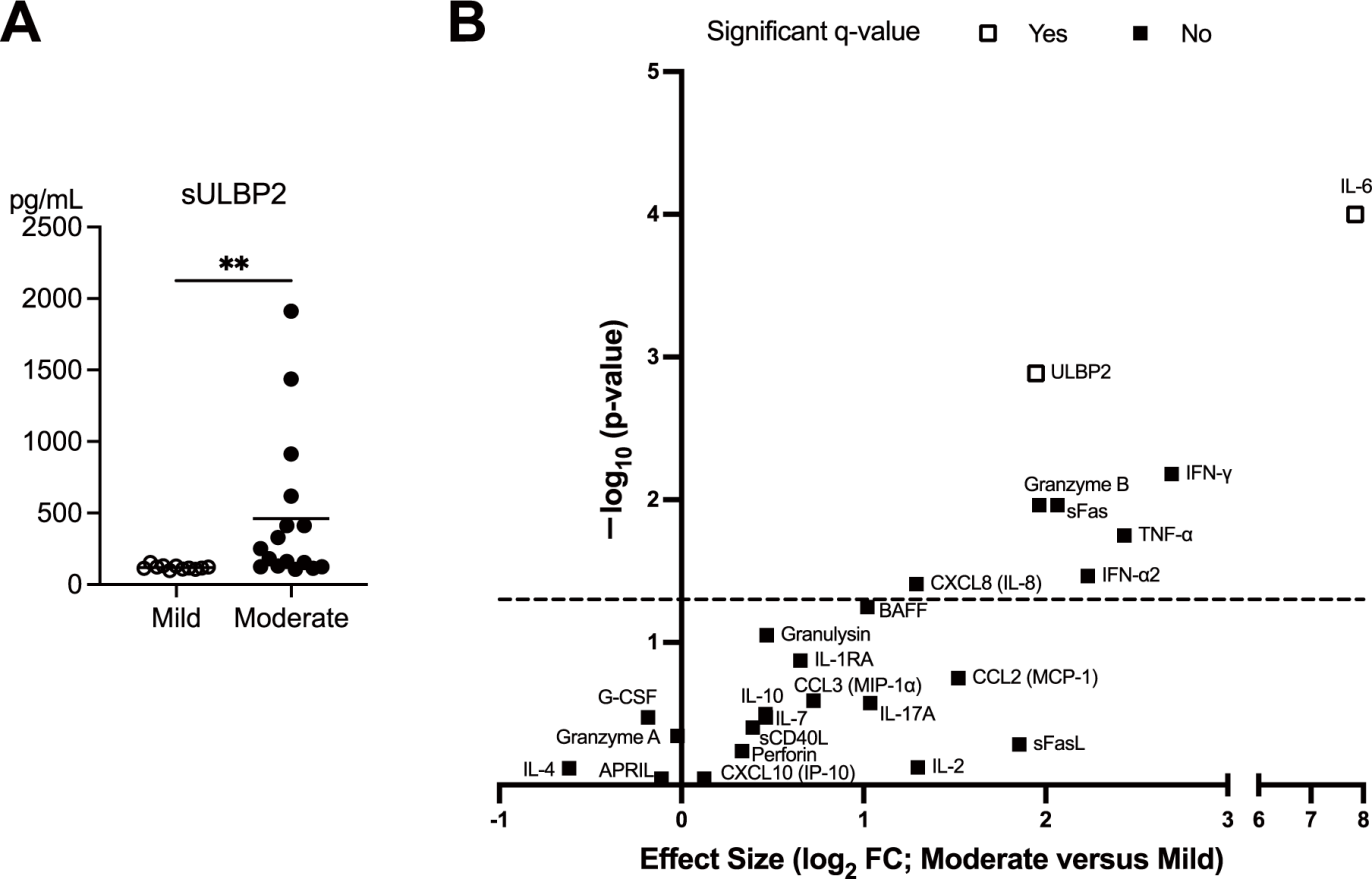
Elevated sULBP2 levels in patients with moderate COVID-19. **(A)** Serum levels of sULBP2 at the clinically most symptomatic time point during hospitalization were measured in the mild (n = 11) and moderate (n = 16) groups using the Human ULBP2 SimpleStep ELISA^®^ Kit. White circles and black circles represent individual patient values for the mild group and moderate group, respectively. Horizontal bars indicate the mean values. Statistical significance is denoted as **p < 0.01. **(B)** A volcano plot generated to compare the mild and moderate cases at the clinically most symptomatic time point using the levels of 24 serum markers and sULBP2. The y-axis represents −log10 (q-value), whereas the x-axis represents log2 fold change (FC), where FC is defined as the mean value in moderate cases divided by the mean value in mild cases. The dashed line represents nominal p = 0.05, indicating that serum markers above the line are significant without multiple comparison correction. Statistical analyses were conducted using the Mann–Whitney U test for comparisons between disease severity group. White squares indicate that the serum markers the remained significant after false discovery rate (FDR) correction performed using the two-stage linear step-up procedure of Benjamini, Krieger, and Yekutieli, whereas the black squares indicate those that were not significant. A threshold of q < 0.05 was considered statistically significant.

The expression of ULBP2, a ligand for the NK cell-activating receptor NKG2D, was assessed by measuring serum sULBP2 levels as a surrogate marker at the clinically most symptomatic time point during hospitalization. Serum sULBP2 levels were significantly higher in the moderate group than in the mild group, suggesting increased ligand expression on infected cell surfaces, potentially enhancing NK cell activation in moderate disease. (**Figure 5A**). To compare mild and moderate cases and evaluate the relative significance of sULBP2 among other immune factors, a volcano plot was generated using the levels of 24 serum markers plus sULBP2 (25 parameters in total) measured at the clinically most symptomatic time point. The analysis identified a significant association of IL-6 and sULBP2 with disease severity (**Figure 5B**).

### 3.7 Correlation analysis among 24 serum markers and sULBP2

**Figure 6:**
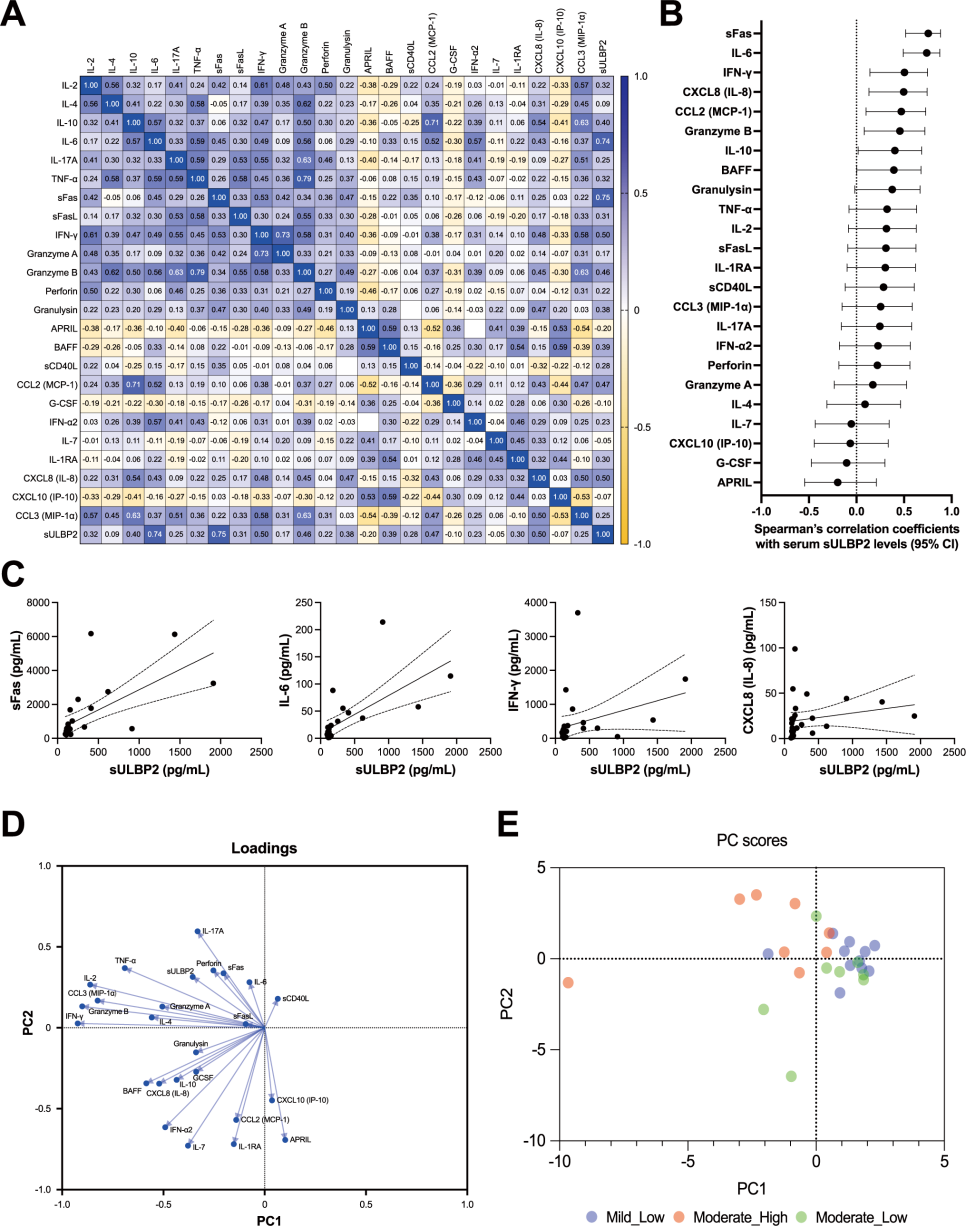
Correlations among cytokines, cytotoxic mediators, and sULBP2 in COVID-19 cases. **(A-C)** Correlation analysis of the serum levels of 24 cytokines, cytotoxic mediators, and soluble marker, along with sULBP2, at the clinically most symptomatic time point during hospitalization in mild (n = 11) and moderate (n = 16) cases. **(A)** Heatmap displaying Spearman’s rank correlation coefficients (Spearman-r). **(B)** Dot plot displaying Spearman’s correlation coefficients and their 95% confidence intervals for the correlations between sULBP2 and the 24 factors. **(C)** Four separate XY scatter plots, showing the relationships between sULBP2 concentrations (x-axis) and IL-6, IFN-γ, sFas, and CXCL8 (IL-8) concentrations (y-axis). These four factors were selected based on the coefficients of their correlations with sULBP2, which were greater than 0.5, as identified in **(A)**. Each plot includes a single linear regression line (solid line) with 95% confidence intervals represented by dashed lines. **(D–E)** Principal component analysis (PCA). **(D)** Loading plot showing the contributions of each factor to the principal components. **(E)** PCA scatter plot of PC-1 versus PC-2. Blue circles represent individual patients in the mild group. Red circles represent patients in the moderate group with higher sULBP2 levels (> 200 pg/mL), and green circles represent patients in the moderate group with lower sULBP2 levels (< 200 pg/mL).

The correlations among the 25 parameters (24 markers plus sULBP2) were analyzed using serum samples collected at the clinically most symptomatic time point of the 27 cases in this study. A heat map of correlation coefficients revealed distinct patterns (**Figure 6A**). Factors primarily associated with NK/CD8^+^ T cells, including IFN-γ, IL-6, CXCL8 (IL-8), sFas, and granzyme B, exhibit positive correlations with each other (blue shades), while showing negative correlations with B cell-associated cytokines, such as APRIL, BAFF, IL-7, and soluble CD40L (sCD40L) (14, 15) (yellow shades). These findings suggest complementary interactions between NK/CD8^+^ T cell-related factors and B cell-related factors. Notably, among all analyzed factors, sULBP2 showed the strongest correlations with sFas and IL-6 (**Figure 6A**, **Supplementary Figures S5A, B**). Factors highly correlated with sULBP2 (correlation coefficients ≥ 0.5) included sFas (r = 0.75), IL-6 (r = 0.74), IFN-γ (r = 0.50), and CXCL8 (IL-8) (r = 0.50) (**Figures 6B, C**).

### 3.8 PCA of immune marker profiles

To further explore the relationships among these 25 parameters (24 markers plus sULBP2), PCA was performed using serum samples from 27 COVID-19 cases at the clinically most symptomatic time point. Parallel analysis identified two principal components (PC1 and PC2) as the main contributors to data variance, collectively accounting for 39.66% of the total variance (PC1: 23.87%, PC2: 15.79%) (**Figure 6D**). PC1 was primarily influenced by cytokines and cytotoxic mediators associated with NK/CD8^+^ T cell activity, including IFN-γ, granzyme B, IL-2, and TNF-α, all of which had strong negative loadings. In contrast, PC2 was primarily driven by factors associated with B cell function and immune regulation, such as APRIL, IL-7, and IL-1RA. The loading of sULBP2 was moderate for both PC1 (−0.356) and PC2 (0.314), aligning with inflammatory mediators such as IL-6, sFas, and IL-17A, while inversely correlated with B cell-associated and regulatory factors, including APRIL, IL-7, and IL-1RA. These findings suggest that sULBP2 is involved in pathways related to both NK/CD8^+^ T cell activation and inflammation, rather than B cell-mediated immune regulation.

To visualize the distribution of sULBP2 levels in relation to PCA scores, a color-mapped PCA score plot was generated using a cutoff value of 200 pg/mL (**Figure 6E**). This threshold was selected to divide the moderate group (n = 16) into two equal subgroups (n = 8 each) for balanced comparisons. Notably, all mild cases had sULBP2 levels below 200 pg/mL. In the PCA score plot, mild cases were primarily distributed in the PC1-positive region, overlapping with moderate cases with lower sULBP2 levels (< 200 pg/mL). Among moderate cases, those with sULBP2 ≥ 200 pg/mL clustered predominantly in the PC1-negative, PC2-positive region, aligning with inflammatory markers such as IL-6 and sFas. In contrast, moderate cases with lower sULBP2 levels tended to be positioned in the PC1-positive, PC2-negative region, indicating a distinct distribution pattern within the moderate group.

## 4 Discussion

In this study, NK cell surface markers and serum cytokine levels were measured at the time of admission and during recovery and were compared between patients with mild and moderate COVID-19 caused by the Omicron variant of SARS-CoV-2. Findings from the analysis of clinical laboratory indicators suggest that the patients with moderate COVID-19 exhibited a more pronounced inflammatory response than those with mild COVID-19, as evidenced by their elevated neutrophil counts and increased levels of inflammatory markers such as CRP and LDH. In addition, flow cytometric analysis revealed that in the patients with mild disease, an increase in the NK cell fraction was observed along with an expansion of unconventional CD56^dim^CD16^-^ NK cells, which displayed increased expression of the activating receptor NKG2D on their surfaces. However, the levels of cytotoxic mediators secreted by NK cells, including granulysin, granzyme A, and granzyme B, were reduced, suggesting that the enhanced NKG2D expression may not necessarily translate into augmented NK cell cytotoxic function. Furthermore, in patients with moderate disease, the elevation of inflammatory markers correlated with increased levels of pro-inflammatory cytokines such as IL-6 and IFN-γ, as well as sULBP2. Because sULBP2 is considered a potential surrogate maker of cell surface ULBP2 that stimulates NK cells through NKG2D, NK cells may be strongly stimulated during viral infection. However, NK cells in these patients exhibited a significant increase in the expression of the inhibitory receptor TIGIT, irrespective of the degree of NKG2D upregulation, implying that NK cell exhaustion may outweigh activation. These findings highlight that in COVID-19, the suppression of innate immunity, specifically NK cell function, is dependent on disease severity: mild cases are characterized by the expansion of unconventional CD56^dim^CD16^-^ NK cell, whereas NK cell exhaustion predominates in moderate cases. However, we cannot exclude the possibility that increased susceptibility to NK cell exhaustion due to age-related immunosenescence may contribute to more severe disease.

**Figure 7:**
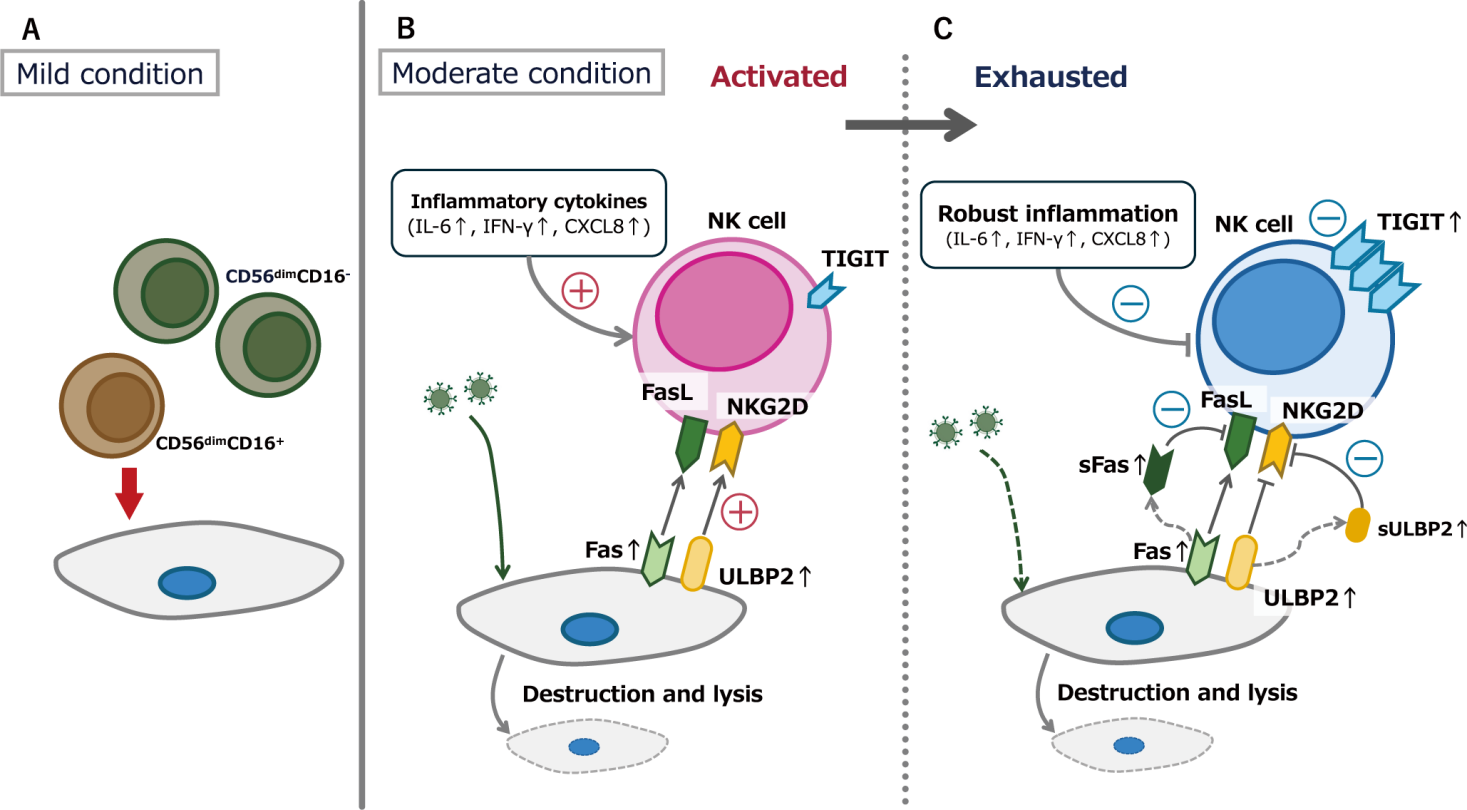
Conceptual figure of the thesis of this research. **(A)** In the patients with mild COVID-19, CD56^dim^CD16^-^ NK cells may exhibit lower cytotoxic activity than the more mature CD56^dim^CD16^+^ NK cells. **(B)** During the acute phase of moderate COVID-19, inflammatory cytokine elevation and increased expression of molecules that activate NK cell function occur simultaneously. **(C)** NK cell exhaustion in moderate cases following NK cell activation. Both CD56^dim^CD16^-^ and CD56^dim^CD16^+^ subsets exhibited upregulated expression of the inhibitory receptor TIGIT, with some cells also showing concurrent upregulation of the activating receptor NKG2D.

CD56^dim^ NK cells are mature NK cells with potent cytotoxic functions, and can be subdivided into CD56^dim^CD16^-^ and CD56^dim^CD16^+^ subsets based on their surface expression of CD16 (16). In the present study, CD56^dim^CD16^-^ NK cells, which were increased at the time of admission in mild cases, represent an intermediate subsets in the NK cell differentiation process, transitioning from CD56^bright^ NK cells to CD56^dim^CD16^-^ NK cells and eventually to CD56^dim^CD16^+^ NK cells. The clinical significance of this subset remains unclear. However, unlike CD56^dim^CD16^+^ NK cells, which predominate under normal conditions, CD56^dim^CD16^-^ NK cells have been reported to increase in proportion during disease states. An increased proportion of CD56^dim^CD16^-^ NK cell has been observed in conditions such as breast cancer (17), infectious mononucleosis (18), and type 1 diabetes mellitus (19). Similarly, elevated CD56^dim^CD16^-^ NK cell counts have been reported in COVID-19 (20, 21), which aligns with the findings of the present study. Notably, previous research has shown that CD56^dim^CD16^-^ NK cells exhibit weak cytotoxicity (22). Furthermore, these cells have been reported to increase in number while exhibiting upregulated expression of inhibitory KIR receptors (21). Based on these characteristics, CD56^dim^CD16^-^ NK cells can be considered to have lower cytotoxic activity than the more mature CD56^dim^CD16^+^ NK cells (**Figure 7A**).

Two key features were observed in the patients with moderate COVID-19 in the present study. The first was a significant elevation in inflammatory cytokine levels, including IL-6, IFN-γ, IFN-α2, and IL-10. Elevated inflammatory cytokines levels in patients with COVID-19 have been documented in several studies, including IL-6 (23), IL-10 (24), IFN-γ (25), IFN-α2 (26), and sFas (20). Furthermore, elevated levels of inflammatory cytokines, particularly IL-6, have been reported to correlate with COVID-19 severity (23, 27). Consistently, in the present study, only patients with moderate COVID-19 exhibited a significant increase in cytokine levels during the acute phase of the disease (at the time of admission). Additionally, our findings confirmed that the increase in sULBP2, which influences NK cell function, correlated with elevation of cytokine levels. Given that sULBP2 is a surrogate marker of ULBP2, which is present on the surfaces of virus-infected cells (8), it can be inferred that the rise in inflammatory cytokine levels and the expression of molecules that activate NK cell function occur simultaneously during the acute phase of moderate COVID-19 (**Figure 7B**) (28, 29).

The second feature of this study is evidence suggesting NK cell exhaustion, characterized by increased TIGIT expression, in patients with moderate COVID-19. In contrast, patients with mild COVID-19 showed no changes in the proportion of TIGIT-expressing cells attributable to SARS-CoV-2 infection. Additionally, TIGIT^+^ cells exhibited high NKG2D expression. In contrast, patients with moderate COVID-19 exhibited increased TIGIT expression, with a notable rise in both TIGIT^+^NKG2D^+^ and TIGIT^+^NKG2D^-^ cells. These findings suggest that NK cell activation and exhaustion coexist during the acute phase of moderate COVID-19, with exhaustion being predominant. This result is consistent with previous reports indicating that NK cells express the inhibitory receptor TIGIT and undergo functional suppression in patients with COVID-19 (30–32). This NK cell exhaustion may be driven by two mechanisms. The first mechanism is exhaustion following activation, driven by elevated levels of cytokines and sULBP2. Some studies have suggested that IL-6 downregulates NKG2D on NK cell surfaces, potentially impairing NK cell activity (33). Similarly, ULBP2 stimulation of NKG2D may lead to NKG2D downregulation on the NK cell surface (34). The second mechanism is that stimulation of NKG2D on NK cells may enhance TIGIT expression (35) (**Figure 7C**). These mechanisms, either independently or in combination, may contribute to elevated cytokine levels in moderate cases of COVID-19, leading to NK cell activation followed by exhaustion.

This study has two distinctive strengths. First, we examined the immunological characteristics of patients with mild-to-moderate COVID-19. Several studies have investigated NK cell alterations in COVID-19 in a pre-Omicron era, including comparisons between mild and severe cases (36) and between moderate and severe cases (37). NK cell phenotypes have also been analyzed in a pooled group of mild-to-moderate cases without distinguishing between them (38). However, direct comparisons between mild and moderate COVID-19 cases remain limited. Second, we longitudinally investigated changes in their immune statuses throughout the disease course. Regarding the first aspect, the significance of analyzing immune function in mild and moderate cases, while excluding severe cases, is as follows. The pathophysiology of severe COVID-19 is characterized by an excessive cytokine storm, leading to extreme and terminal conditions. This phenomenon was a major contributor to the strain on healthcare systems and the high mortality rates observed from the early pandemic period to the delta variant period. Consequently, although some studies have compared severe cases with mild or moderate disease, numerous studies have focused on comparisons between patients with severe COVID-19 and healthy individuals (39). However, in the current Omicron-dominant era, mild and moderate cases constitute the majority of patients, making it essential to understand the subtle immunological changes that precede severe disease progression. Previous studies have reported reduced NK cell counts and impaired cytolytic activity in severe COVID-19 (33, 40, 41). However, in the present study, moderate COVID-19 cases exhibited an increase in NK cell numbers, along with simultaneous activation and exhaustion, highlighting differences from findings reported in severe COVID-19. These observations suggest that the immune alterations observed in this study may represent an intermediate stage in the progression toward severe COVID-19, which is characterized by NK cell depletion and exhaustion. As for the second aspect, most previous studies investigating immune function in COVID-19 have focused on cross-sectional comparisons between different groups, such as healthy individuals and patients with severe COVID-19 (42–44), while longitudinal analyses tracking temporal changes within the same group remain insufficiently reported. Capturing immune changes from disease onset to recovery is crucial for elucidating the correlation between the immune response dynamics to SARS-CoV-2 infection and the clinical course of the disease. However, the existing longitudinal studies are constrained by small sample sizes (45) or focus primarily on changes in immune cell counts, such as neutrophils, monocytes, and lymphocytes, and antibody levels (46). Moreover, only a few studies included longitudinal evaluation of intercellular communication via cell surface markers. Therefore, the present study provides new insights into the immunological changes that occur following COVID-19 infection and contributes to a deeper understanding of immune mechanisms in the disease course.

This study has several limitations. First, as all patients developed symptomatic COVID-19, data from the asymptomatic phase of the disease were not collected. Consequently, it was not possible to accurately determine whether the significant decrease in certain parameters from admission to recovery reflects a return to normal levels after an initial increase at the time of hospitalization. Second, some patient characteristics and clinical factors were not balanced between the two groups. In this study, sex, age, and certain comorbidities such as obesity and chronic kidney disease, as well as treatment regimens, were not evenly distributed between the groups. Regarding sex, previous studies have suggested that it may influence immune responses and disease severity in SARS-CoV-2 infection (47), and this potential influence should be considered when interpreting our findings. In addition, the older age and greater comorbidity burden in the moderate group may have contributed to the elevated levels of inflammatory and cytotoxic mediators observed in these patients. These factors could have influenced baseline levels of immune activation (48, 49) and the magnitude of the inflammatory response to SARS-CoV-2 infection (50, 51). Due to the limited sample size, stratified analyses to disentangle these potential influences were not feasible in this study. Therefore, future prospective studies with well-matched cohorts are warranted to validate our findings. Third, the small sample size (n = 27) limited the statistical power of the results. Additionally, the retrospective design of the study and the inclusion of only hospitalized patients may have introduced selection bias. Furthermore, as this was a single-center cohort study, the generalizability of the results to other populations is limited. Finally, we conducted the analyses using lymphocytes from cryopreserved PBMCs, which may have affected cell surface marker expression due to the freezing process. Future studies conducted using samples analyzed immediately after collection are required. However, it should be noted that immediate analysis after blood draw could potentially introduce inter-assay variability in flow cytometry.

In conclusion, this study investigated alterations in NK cell-associated immunity dynamics in patients with mild and moderate COVID-19 who were hospitalized during the Omicron phase of the COVID-19 pandemic. The results suggest that in patients with mild COVID-19, NK cell cytotoxicity might be suppressed in association with an increase in unconventional CD56^dim^CD16^-^ NK cells, whereas in patients with moderate COVID-19, elevated serum levels of inflammatory cytokines and sULBP2 might be associated with NK cell activation and functional exhaustion. These findings indicate that while NK cell cytotoxic activity appears to be impaired in COVID-19, the underlying mechanisms might differ depending on disease severity. Further studies are needed to clarify the role of NK cells in COVID-19 pathophysiology.

## Data Availability

The raw data supporting the conclusions of this article will be made available by the authors, without undue reservation.
