# Supplementary material for: Distinct immunity dynamics of natural killer cells in mild and moderate COVID-19 cases during the Omicron variant phase
Source: Front Immunol. 2025 May 12;16:1594296. doi: 10.3389/fimmu.2025.1594296 (PMC12104262; doi:10.3389/fimmu.2025.1594296)
Supplement: Supplementary file 1 [file DataSheet1.docx]

Supplementary Material

# Supplementary Methods

1.1 Study design and eligibility criteria

This retrospective observational cohort study was conducted at Tottori University Hospital between January and May 2022, during the Omicron phase of the COVID-19 pandemic. The sample size was not predetermined as all eligible patients with available data during the study period were included. Patients aged 16 years or older who were hospitalized for confirmed SARS-CoV-2 infection were eligible for inclusion. COVID-19 severity was classified as mild, moderate, or severe based on the Japanese clinical practice guideline. Patients with mild COVID-19 were defined as asymptomatic patients or those who presented with only respiratory symptoms. Patients with moderate COVID-19 were defined as those with dyspnea or radiological evidence of pneumonia (with or without oxygen requirement). Patients with severe COVID-19 were defined as those who required admission into the intensive care unit or mechanical ventilation. Patients were excluded if they lacked sufficient samples required for the evaluations performed in this study, if they were diagnosed with severe COVID-19, or if the principal investigator deemed them ineligible based on clinical or other relevant considerations.

1.2. *Description of the patient selection process*

A total of 75 patients met the eligibility criteria and were enrolled in this study. Of these, 50 provided written informed consent, whereas 25 provided consent through an opt-out approach. Four patients were excluded because their recovery-phase blood samples were unavailable, and three severe cases were also excluded as per the study design, leaving 68 eligible patients (52 with mild COVID-19 and 16 with moderate COVID-19. To ensure a well-balanced comparison for cost-effectiveness of the experiments, 11 patients with mild COVID-19 were manually selected to match the number of patients with moderate disease. Selection was based on clinical data from medical records, forcusing on comorbidities and the use of immunosuppressive drugs. Twenty-seven patients, including 11 and 16 with mild and moderate COVID-19, respectively, were included in the final analyses. The serum cytokine levels of all 27 patients were analyzed to evaluate immunity dynamics over time. One patient was excluded from the analysis of PBMCs owing to technical issues, leaving a final dataset of 26 patients included in the PBMCs analysis. No patient opted out during the study period.

**2. Supplementary Figures**


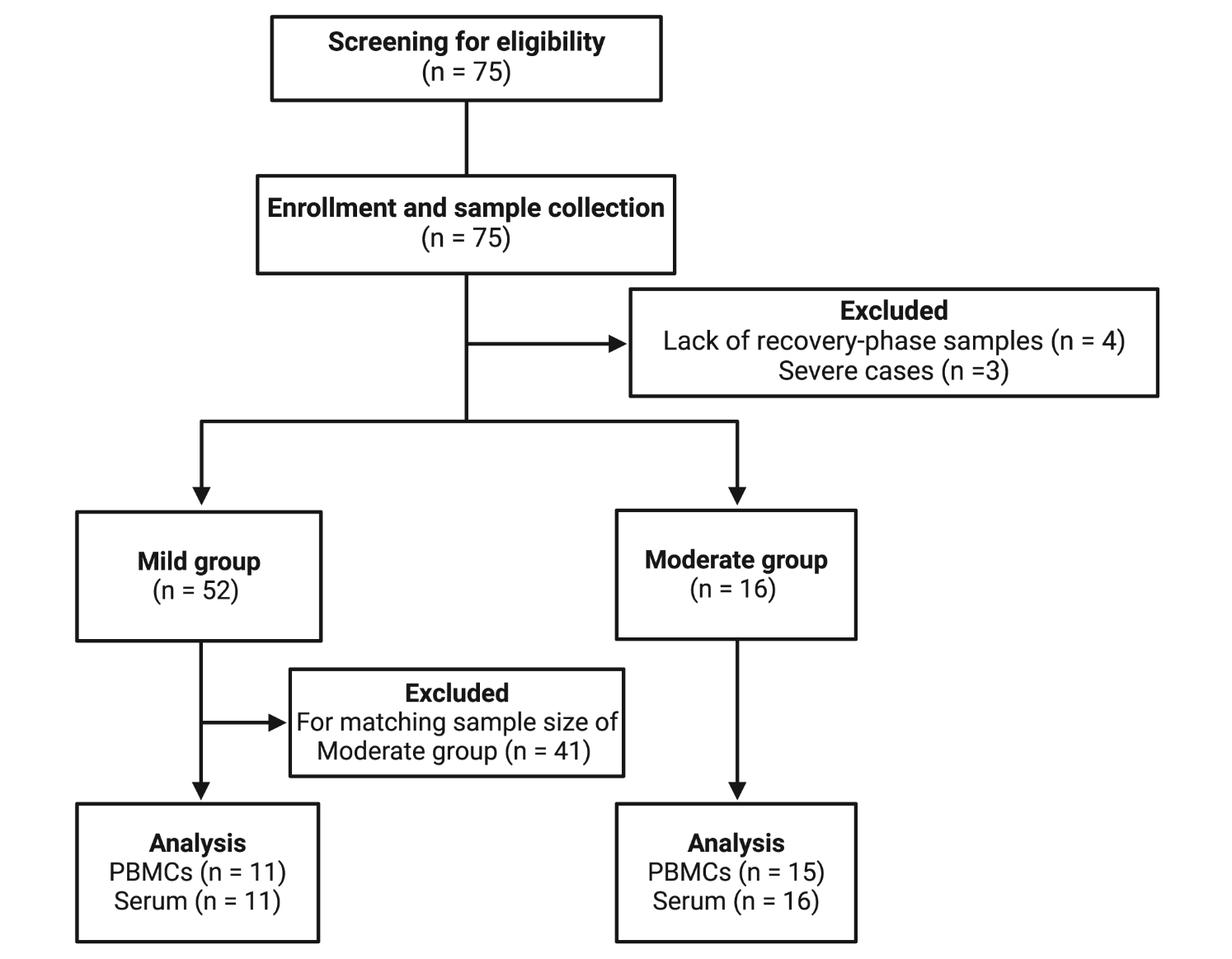


**Supplementary Figure S1.** Patient selection flowchart (Created with BioRender.com)


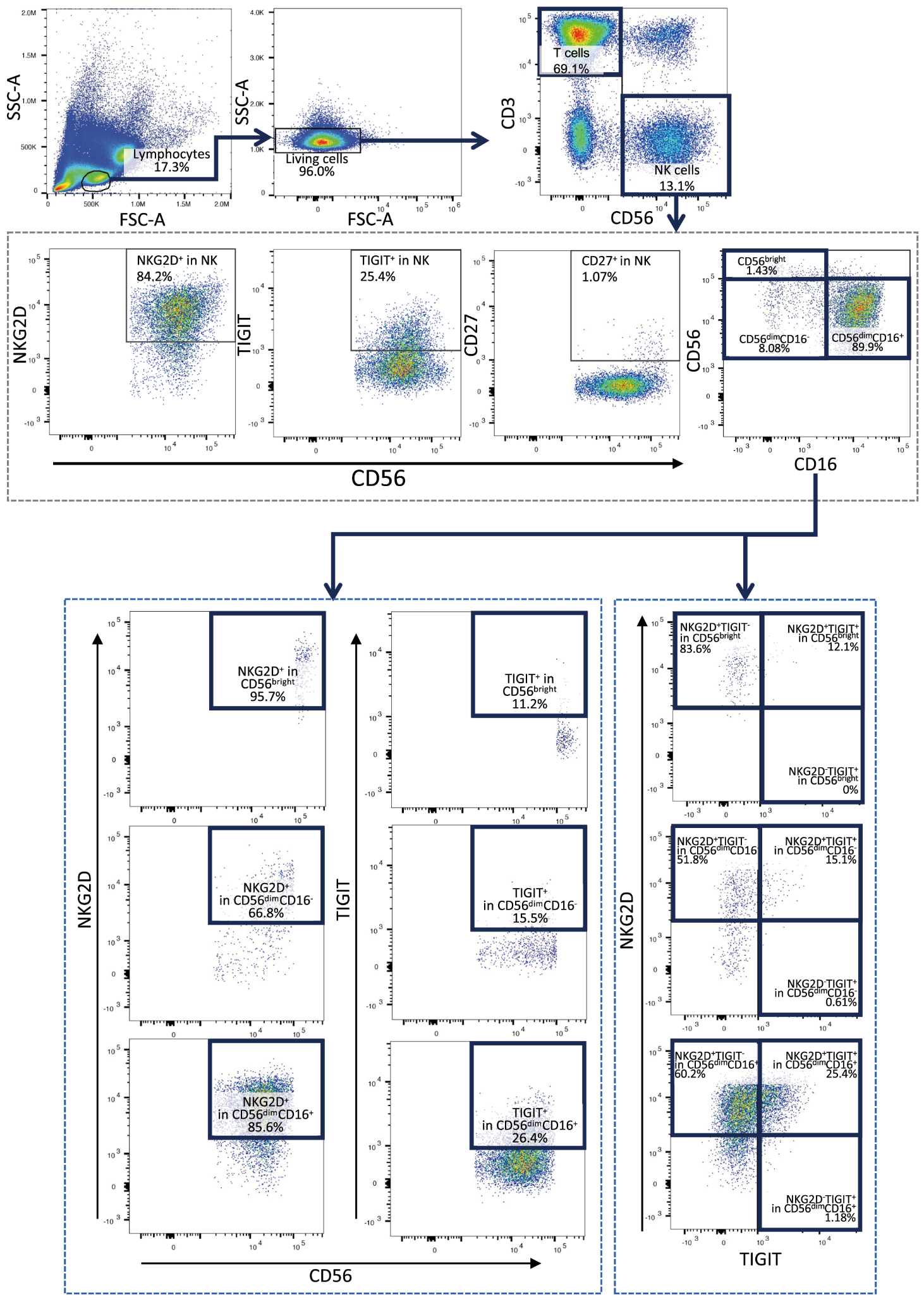
 **Supplementary Figure S2.** The gating strategy for cytometric analysis of NK cells in PBMC samples. NK cells were identified as CD3⁻CD56⁺ cells in the FSC-SSC lymphocyte gate. Fixable Viability Dye eFluor^TM^ 780 was used to detect dying cells. Surface expression of CD3, CD27, CD56, CD16, NKG2D, and TIGIT in NK cells was analyzed using flow cytometry.


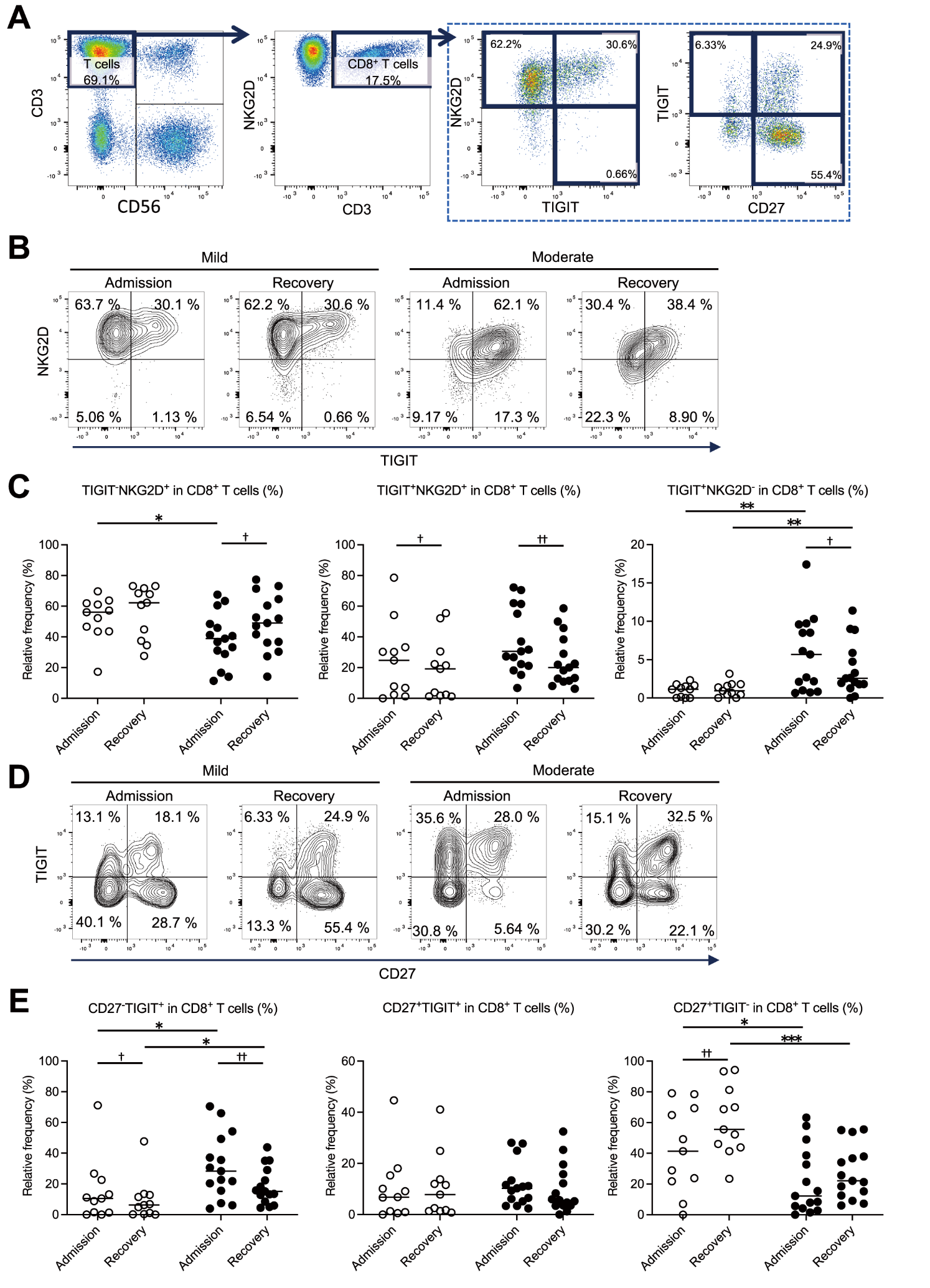


**Supplementary Figure S3.** Correlations among NKG2D, TIGIT, and CD27 expression levels in CD8⁺ T cells in patients with mild and moderate COVID-19. Flow cytometry of PBMCs was performed to generate two-dimensional plots. The proportions measured at the time of admission and during recovery were compared between the mild and moderate groups, highlighting differences related to disease severity. (A) Gating strategy. (B) Representative contour plots of CD8⁺ T cells, with TIGIT on the x-axis and NKG2D on the y-axis. (C) Proportions of TIGIT^-^NKG2D⁺, TIGIT⁺NKG2D⁺, and TIGIT⁺NKG2D⁻ cells among the CD8⁺ T cells. (D) Representative contour plots of CD8⁺ T cells, with CD27 on the x-axis and TIGIT on the y-axis. (E) Proportions of CD27⁻TIGIT⁺, CD27⁺TIGIT⁺, and CD27⁺TIGIT⁻ cells among the CD8⁺ T cells. In (C) and (E), the white and black circles represent individual patient data for the mild group and moderate group, respectively, with the horizontal bars indicating median values. The signed-rank test was used for comparisons between the admission and recovery phases, whereas the Mann-Whitney U test was used for comparisons between disease severity groups. Significance levels were set as follows: † p < 0.05, †† p < 0.01 for the Wilcoxon signed-rank test, * p < 0.05, ** p < 0.01, *** p < 0.001 for the Mann–Whitney U test. Non-significant differences are not marked.


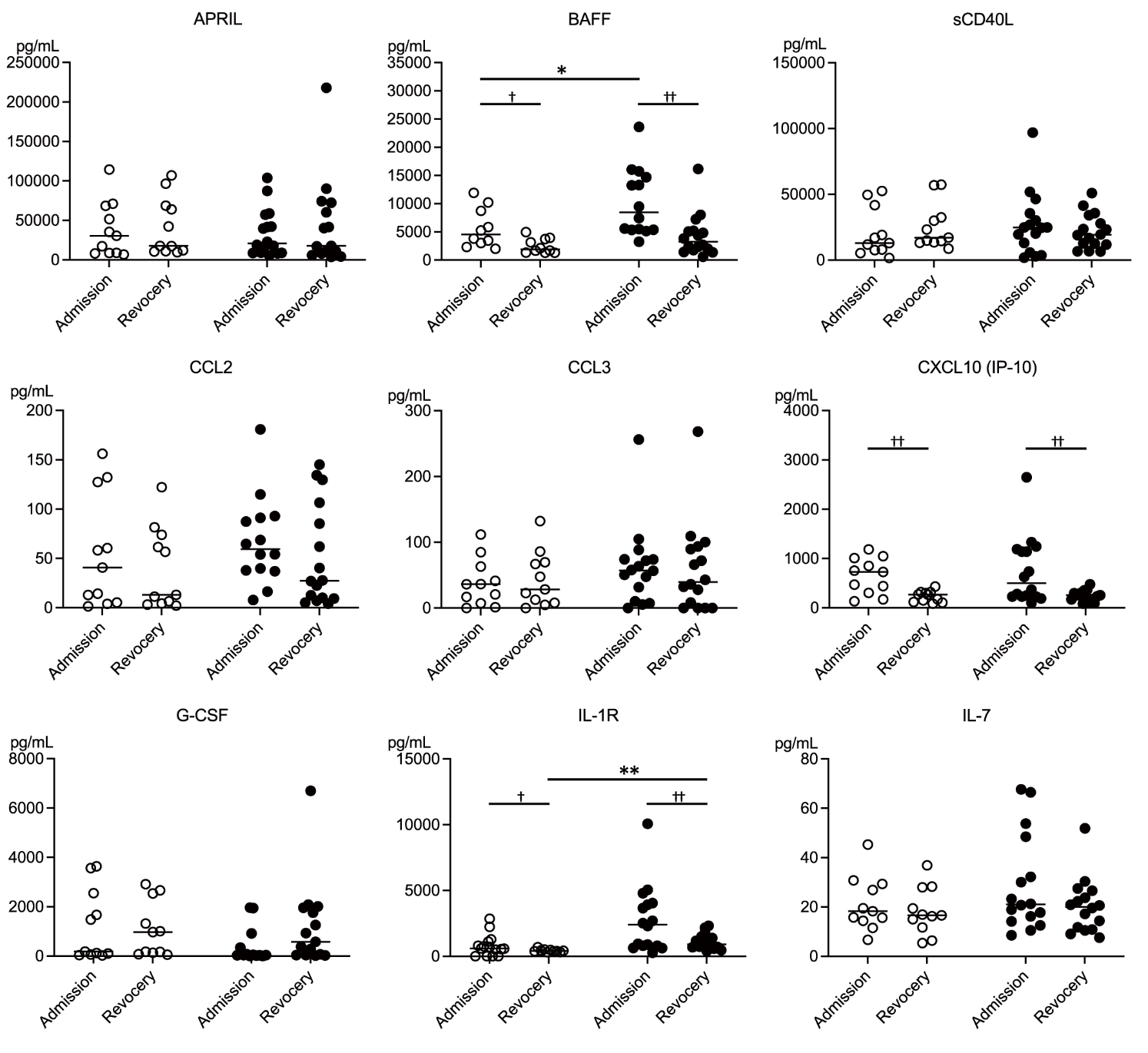


**Supplementary Figure S4**. Cytokine and soluble marker profiles in patients with mild and moderate COVID-19. Serum levels of nine cytokines and soluble markers measured at the time of admission and during recovery were compared between the mild (n = 11) and moderate (n = 16) groups. The white and black circles represent individual patient data for the mild and moderate groups, respectively, and the horizontal bars indicate median values. The Wilcoxon signed-rank test was used for comparisons between the admission and recovery phases, whereas the Mann–Whitney U test was used for comparisons between disease severity groups. Significance levels were set as follows: † p < 0.05, †† p < 0.01 for the Wilcoxon signed-rank test, * p < 0.05, ** p < 0.01 for the Mann–Whitney U test. Non-significant differences are not marked.

**
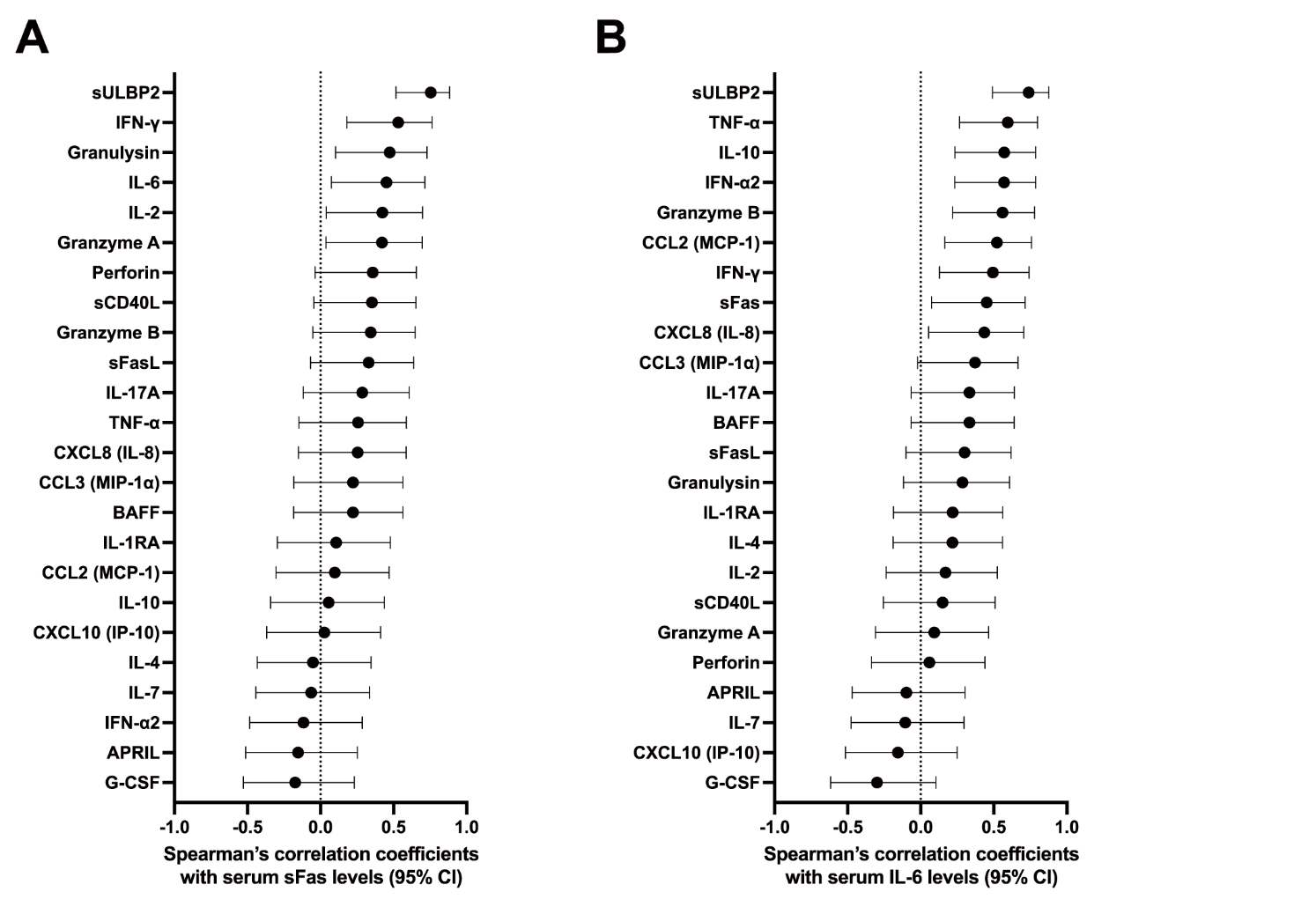
**

**Supplementary Figure S5**. Correlation analysis of the serum levels of 24 cytokines, cytotoxic mediators, and soluble markers, along with sULBP2, at the clinically most symptomatic time point during hospitalization in mild (n = 11) and moderate (n = 16) COVID-19 cases. Dot plots display Spearman’s correlation coefficients and 95% confidence intervals for the correlations of sFas (A) and IL-6 (B) with the other 24 factors.
